# Nutrigenetics and Metabolic Disease: Current Status and Implications for Personalised Nutrition

**DOI:** 10.3390/nu5010032

**Published:** 2013-01-10

**Authors:** Catherine M. Phillips

**Affiliations:** HRB Centre for Diet and Health Research, Department of Epidemiology and Public Health, University College Cork, College Road, Cork, Ireland; E-Mail: c.phillips@ucc.ie; Tel.: +353-21-490-5538; Fax: +353-21-420-5469

**Keywords:** nutrigenetics, metabolic health, dietary fat, obesity, insulin resistance, metabolic syndrome, diabetes, metabotype, gene-nutrient interaction

## Abstract

Obesity, particularly central adiposity, is the primary causal factor in the development of insulin resistance, the hallmark of the metabolic syndrome (MetS), a common condition characterized by dyslipidaemia and hypertension, which is associated with increased risk of cardiovascular disease (CVD) and type 2 diabetes (T2DM). Interactions between genetic and environmental factors such as diet and lifestyle, particularly over-nutrition and sedentary behavior, promote the progression and pathogenesis of these polygenic diet-related diseases. Their current prevalence is increasing dramatically to epidemic proportions. Nutrition is probably the most important environmental factor that modulates expression of genes involved in metabolic pathways and the variety of phenotypes associated with obesity, the MetS and T2DM. Furthermore, the health effects of nutrients may be modulated by genetic variants. Nutrigenomics and nutrigenetics require an understanding of nutrition, genetics, biochemistry and a range of “omic” technologies to investigate the complex interaction between genetic and environmental factors relevant to metabolic health and disease. These rapidly developing fields of nutritional science hold much promise in improving nutrition for optimal personal and public health. This review presents the current state of the art in nutrigenetic research illustrating the significance of gene-nutrient interactions in the context of metabolic disease.

## 1. Introduction

The MetS represents a constellation of metabolic perturbations including central obesity, insulin resistance, dyslipidaemia characterized by raised triglyceride and reduced high density lipoprotein concentrations and hypertension. The MetS and these interrelated risk factors are associated with increased risk of type 2 diabetes (T2DM) and cardiovascular disease (CVD) [1]. Numerous definitions of the MetS have been proposed; initially by the WHO in 1998 [2] and subsequently by the European Group for the Study of Insulin Resistance in 1999 [3], the National Cholesterol Education Program’s Adult Treatment Panel III report (NCEP ATP III) in 2001 [4] and in 2005 by the International Diabetes Federation (IDF) [5] and the IDF in conjunction with the American Heart Association and the National Heart, Lung and Blood Institute [6]. While details differ between definitions, all agree on the essential components (central obesity, insulin resistance/glucose intolerance, dyslipidaemia and hypertension). Notwithstanding the varying definitions it is clear that the incidence of the MetS is increasing among men and women of all ages and ethnicities [7]. Recent estimates from the US show that the prevalence of the MetS among adults ranges from 34.3% to 38.5% depending on the criteria used to define abdominal obesity [7]. Thus it is conceivable that 77 to 86 million adults in the US meet current MetS criteria. Individuals with the MetS have a five-fold increased risk of developing T2DM. Coupled with this is a two-fold risk of developing CVD over the next 5 to 10 years compared to individuals without the syndrome. Lifetime risk is even higher. The prevalence of obesity is also increasing worldwide, with the condition predicted to affect more than one billion people by the year 2020 [8]. Obesity and weight-gain are directly related to T2DM risk [9]. Excess adiposity, particularly central adiposity, is a key causal factor in the development of insulin resistance, the hallmark of the MetS. The increasing global prevalence of T2DM in children and adults, and its medical and socio-economic consequences represent a major public health concern. Recent estimates predict in excess of 400 million individuals with T2DM worldwide by the year 2030 [10]. 

Complex gene-environment interactions are certainly contributing to the current diabesity epidemic. Family and twin studies indicate that up to 80% of the variance in body mass index (BMI) is attributable to genetic factors. Genetic factors also contribute approximately 50% towards T2DM risk. Heritability rates of 10%–30% for the MetS have been estimated [11,12], indicating that these conditions are partly heritable. Nutrition and physical activity are key environmental factors, which potentially interact with genetic predisposition, to promote the progression and pathogenesis of these combined environmental and polygenic, diet-related diseases. Excessive caloric intake and sedentary lifestyle promote the obese phenotype. More than half of adults in Europe and the US are overweight or obese, leading to the MetS, which in turn greatly enhances subsequent risk of cardiometabolic disease [13]. There is no doubt that a genetic component can also impact on the risk of insulin resistance, the sensitivity to which may be further amplified by poor diet. If we have a greater understanding of potential gene-nutrient interactions, then it may be possible to manipulate diet in such a way to minimize the metabolic risk of obesity, to attenuate insulin resistance and development of cardiometabolic disease. At a public health level, more attention must be given to modification of lifestyles of the general public to reduce risk of obesity and T2DM and to increase physical activity. At a clinical level, individual patients with increased metabolic risk need to be identified so that their multiple risk factors can be reduced. Early identification of “at risk” individuals is of paramount importance. Considering the long asymptomatic period often preceding the manifestation of T2DM and CVD, early diagnosis could enable earlier targeted interventions such as implementation of healthy lifestyle changes in nutritional behavior and exercise or pharmacotherapy, thus reducing disease development. From the nutritional/dietary advice perspective, this review will focus on dietary fatty acids, as these are the most energy dense nutrients. A deeper understanding of the underlying gene-nutrient interactions, to aid an understanding of the link between nutrition and health, will provide the evidence base to define whether more targeted nutritional advice is an appropriate public health approach. Nutrigenetics hold much promise in terms of both public health nutrition and for individuals and genetic subgroups. In this review we will present the current state of the art, illustrating the significance of gene-nutrient interactions in the context of diet-related metabolic disease. 

## 2. Dietary Fat and Metabolic Health

Dietary fat is an important environmental factor, wherein excessive exposure plays a key role in the development of the MetS [14,15,16,17,18]. High-fat diets, in particular high saturated fatty acid (SFA) diets, have been shown to exert detrimental effects on adiposity, inflammation and insulin sensitivity, promoting the development of insulin resistance, the MetS and T2DM [17,19,20,21]. Diets rich in monounsaturated fatty acids (MUFA) have been associated with improvements in insulin sensitivity in healthy subjects [22]. In the LIPGENE study, a large pan-European isocaloric dietary intervention study of MetS subjects, substitution of SFA with either MUFA or low-fat, high-complex carbohydrate to improve insulin sensitivity was only effective in individuals whose habitual pre-intervention dietary fat intake was below the median (<36% energy from fat) [23]. Evidence from the MUFA in Obesity (MUFObes) study suggest that MUFA rich diets had beneficial effects on insulin and glucose concentrations and were associated with reduced body fat regain [24,25]. The KANWU study showed that a high-SFA diet reduced insulin sensitivity in overweight subjects, and post-hoc analysis indicated that isoenergetic substitution of SFA for MUFA improved insulin sensitivity but only in subjects whose habitual pre-intervention dietary fat intake was below the median [26]. While cell and animal studies have demonstrated beneficial effects of long chain *n*-3 polyunsaturated fatty acids (LC *n*-3 PUFA) on inflammation and insulin sensitivity [27,28,29] translation of these potentially anti-diabetic effects in humans has proven more difficult with conflicting epidemiological data in relation to their effect on insulin resistance in humans [23]. A limited number of large human dietary intervention studies have been performed to determine the effects of dietary quantity and quality on risk factors associated with metabolic health. Considering that up to 30% of subjects are not responsive to interventions, variable intervention outcomes are perhaps not surprising. Moreover it seems that pre-intervention dietary intake may affect outcome. A range of methods to assess dietary intake including food-frequency questionnaires and weighed or estimated dietary records are currently used. Each has advantages, practical limitations and associated errors. In the context of dietary fatty acids the use of biomarkers of habitual dietary fat intake, such as plasma fatty acids, offers some advantages over self-reported food-frequency questionnaires whereby they are not subject to misclassification of exposure, due to deficiencies in nutrient databases, accuracy of memories or willingness to divulge these details [30]. In contrast to dietary fat measurement, plasma fatty acid composition reflects the combination of dietary fat consumption and endogenous *de novo* fatty acid biosynthesis and metabolism, thus making direct comparisons between some plasma and dietary fatty acid measurements difficult.

The failure of current dietary guidelines in combating the obesity epidemic provides further evidence that the optimal dietary fat composition (amount and type of fatty acid) for optimal metabolic health is still unknown and that the traditional one size fits all approach does not work in the context of obesity and metabolic health. Indeed such inter-individual differences in response to dietary factors and interventions highlight the role of genetics and the potential of a nutrigenetics approach based on identification of nutrient sensitive or responsive genotypes, whereby nutrient intake is manipulated or optimized based on an individuals’ genetic profile to reduce disease risk or improve effectiveness of dietary recommendations. Current evidence to support the nutrigenetics concept with respect to obesity, the MetS and T2DM is largely based on data relating to dietary fat [15,31,32,33] and is discussed in more detail later. 

## 3. Genetic Determinants of Metabolic Disease

It is commonly accepted that the current global diabesity pandemic is being driven by an obesogenic environment which promotes consumption of energy-dense foods and discourages energy expenditure, inevitably leading to an energy imbalance favoring energy storage and weight gain. A number of factors including industrialization and modernization of the built environment (reliance on car use, reduced manual labor, lack of safe pathways or cycle lanes, proximity to fast food and convenience outlets, increased commuting time, *etc*.) as well as the social environment (socioeconomic status, advertising, consumer pressure, *etc*.) have led to more sedentary lifestyle behaviors and freely available caloric abundance. However, the familial clustering associated with obesity is not just due to common environmental factors. Studies of twins, adoptees and families indicate that up to 80% of the variance in BMI is attributable to genetic factors. High relative risk ratios [34] and concordance rates for monozygotic twins compared to dizygotic twins have also been estimated for obesity [35]. Interestingly adoption studies revealed that adoptees’ weight is more similar to that of the biological parents than the adoptive parents [36]. Heritability rates of 25%–40% for BMI and body fat [36,37,38] and 10%–30% for the MetS have been estimated [11,12], indicating that these conditions are partly heritable. Genetic factors are also estimated to contribute approximately 50% towards T2DM risk. Family studies have demonstrated that first degree relatives of T2DM individuals are approximately 3 times more likely to develop the disease than individuals without a positive family history [39,40]. Furthermore, twin studies have shown that concordance rates for monozygotic twins, ranging from 60% to 90%, are significantly higher than those for dizygotic twins. Thus, it is clear that genetic differences between individuals also play a role in the risk of becoming obese and developing the MetS and T2DM. 

### 3.1. Identification of Genes Associated with Metabolic Disease

Monogenic disorders account for up to 5% of all cases of obesity and also diabetes. Over the past 15–20 years, mutations in a number of genes including leptin (*LEP*), leptin receptor (*LEPR*), pro-opiomelanocortin (*POMC*) and melanocortin-4 receptor (*MC4R*) have been associated with monogenic obesity [41]. Six genes account for the majority of the monogenic forms of diabetes: hepatic nuclear factor 4α (*HNF-4α*), glucokinase (*GCK*), hepatic nuclear factor 1α (*HNF-1α*), insulin promoter factor-1 (*IPF-1*), hepatic nuclear factor 1β (*HNF-1β*) and neuro D1 transcription factor (*NEUROD1*) [40]. However, for most individuals genetic predisposition to metabolic disease has a polygenic basis. The absence of large single gene effects and the detection of multiple small effects support this notion, which suggests that only in combination with other predisposing variants does a sizeable phenotypic effect arise. In addition, this implies that certain sets of polygenic variants relevant to these conditions in one individual may not be the same in another individual. Identifying genes associated with any complex trait involves a range of experimental strategies including positional cloning using genome-wide linkage analysis, candidate gene association and more recently genome-wide association studies (GWAS). Genome scanning in several different ethnic groups has identified a number of chromosome regions harboring T2DM and obesity susceptibility genes. Calpain 10 (*CAPN10*), which encodes cysteine protease calpain 10, was the first T2DM susceptibility gene identified through a genome-wide scan followed by positional cloning [42]. Genetic and functional data indicate that *CAPN10* plays an important role in insulin resistance and intermediate phenotypes [43]. *CAPN10* variants have been linked with several MetS phenotypes including hypertriglyceridaemia, BMI and hypertension [44,45,46]. With the advent of high-throughput genetic analysis and the completion of the Human Genome Project our understanding of the genetic architecture and biology of obesity, MetS, insulin resistance and T2DM is improving. GWAS represent a powerful approach to the identification of genes involved in common polygenic diseases. Typically, these studies, which are performed without any prior knowledge regarding the nature or location of the causative genes, analyze thousands of SNPs across the entire genome and involve very large subject numbers. Patterns of association between genotypes and disease status are then identified and evaluated statistically. 

### 3.2. GWAS and Metabolic Health Relevant Loci

In the last six years GWAS have identified more than 50 loci, many of which are novel, relevant to obesity and diabetes. 2006 heralded the identification of the most important T2DM susceptibility gene known so far, transcription factor 7-like 2 (TCF7L2). Two novel single nucleotide polymorphisms (SNP) in the Wnt signaling regulated TCF7L2 gene were associated with increased T2DM risk [47,48], most likely through defective beta-cell function and impaired insulin secretion [47]. Several large studies subsequently replicated and confirmed the association with T2DM risk in various populations and the TCF7L2 rs7903146 SNP has emerged as one of the most important T2DM susceptibility gene variants known to date [49,50,51,52,53,54]. TCF7L2 polymorphisms have also been associated with MetS components such as dyslipidemia and waist circumference [55,56]. However, prospective and population-based MetS association studies have produced conflicting results; with some reporting an association with MetS, hyperglycaemia, impaired insulin secretion and hypertriglyceridaemia [56,57], whilst others found no association with MetS or insulin resistance [58,59]. More recently, TCF7L2 rs7903146 was associated with increased MetS risk, arising from their impaired insulin sensitivity, greater insulin resistance, increased abdominal obesity and hypertension [60]. This association has also been confirmed in a recent systematic review [61]. Following the identification of TCF7L2, previously unknown genetic variants in the fat mass and obesity-associated (*FTO*) gene on chromosome 16 were also linked to T2DM risk through an effect on BMI [62], such that the 16% of adults homozygous for the rs9939609 “risk” A allele were 3 kg heavier and had 1.7 fold increased risk of obesity relative to the homozygous non-risk allele carriers. It has been suggested that the impaired satiety, greater food intake and more frequent loss of eating control reported by individuals with at least one risk allele may account for the observed increased obesity risk [63,64,65]. Importantly, researchers have demonstrated increased obesity risk associated with this polymorphism from childhood into old age [62]. A number of large studies replicated and confirmed the association with obesity risk in European populations [66,67,68] and the *FTO* rs9939609 SNP is now recognized as one of the most important gene variants predisposing to obesity. A recent systematic review on the genetics of the MetS confirmed increased MetS risk with FTO [61]. Other obesity susceptibility loci identified by GWAS include *SHRB1* and *BDNF* [69,70]. Meta-analysis of 21 GWAS cohorts (Meta-Analyses of Glucose and Insulin-related traits Consortium [MAGIC]), identified associations between a number of SNPs in 8 loci (including the candidate genes *ADCY5*, *FADS1*, and *GLIS3*) and fasting glucose concentrations, and also between SNPs in one locus (with the candidate gene *IGF1*) with both fasting insulin levels and insulin resistance [71]. More recently, a joint meta-analysis investigating whether genes involved in insulin resistance pathways could be discovered by accounting for differences in BMI and interactions between BMI and genetic variants identified eight SNPs in six loci (including the candidate genes *COBLL1*/*GRB14*, *IRS1*, *PPP1R3B*, *PDGFC*, *UHRF1BP1*, and *LYPLAL1*) that were associated with fasting insulin, high triglyceride and low high-density lipoprotein levels, suggesting a new series of pathways to identify genes with contributions to multiple phenotypes [72]. It is clear that the contribution of GWAS cohorts to such consortia will provide increased power to detect variants associated with measures of glucose homeostasis, insulin resistance, obesity, T2DM and MetS phenotypes. 

### 3.3. Lipid Metabolism Genes

Dyslipidaemia is one of the very early features of an obese or MetS phenotype and frequently precedes metabolic disturbances in insulin and glucose homeostasis. Both fasting and postprandial lipid metabolism are disturbed in the MetS [73] and in T2DM, particularly among individuals with poor metabolic control [74]. A number of recent reviews present the evidence linking candidate genes with modulation of postprandial lipid metabolism [75,76]. To summarize, genes of note include the apolipoprotein *APOA1*/*C3*/*A4*/*A5 cluster*, *APOB*, *APOE*, *cholesterol ester transfer protein*, *hepatic and lipoprotein lipases*, *TCF7L2*, *glucokinase regulatory protein*, *fatty acid binding proteins*, *microsomal triglyceride transfer protein*, *peroxisome proliferator-activated receptor-γ* (*PPARγ*), *scavenger receptor class B type 1* (*SCARB1*) and *perilipin* (*PLIN*). Prior to the GWAS era the lipid sensitive transcription factor *PPARγ* Pro12Ala polymorphism was identified as the most widely reproduced genetic variation for T2DM risk [39]. The original study investigating the polymorphism in T2DM demonstrated that the alanine allele of this polymorphism was associated with lower BMI, improved insulin sensitivity and thus reduced diabetes risk by 75% [77]. Inconsistent results from subsequent association studies prompted a meta-analysis which confirmed a modest (1.25-fold) but significant increase in diabetes risk for the Pro12Pro genotype [78]. The Pro12Pro genotype also predicts conversion from impaired glucose tolerance to T2DM, with a 3 fold increased T2DM risk in the Pro12 homozygotes compared to 12Ala carriers [79]. In obese subjects diabetes risk is almost doubled among the Pro12 allele carriers. Interestingly the obese phenotype seems to exacerbate the detrimental effect of the *PPARγ* Pro12 allele on insulin sensitivity [80]. Perhaps more importantly is that this variant is very common in most populations. Approximately 98% of Europeans carry at least one copy of the Pro allele, so it is reasonable to speculate that this SNP contributes to a considerable proportion of T2DM risk. In the LIPGENE-SU.VI.MAX study we identified a number of novel associations between SNPs of genes involved in fatty acid and lipoprotein metabolism, including long-chain acyl CoA synthetase 1 (*ACSL1*), acetyl-CoA carboxylase β (*ACC2*), apolipoprotein A-I (*APOA1*) and apolipoprotein B (*APOB*) and lipoprotein lipase (*LPL*) with risk of the MetS or its phenotypes [81,82,83,84]. 

### 3.4. Pro-Inflammatory Cytokines and Other Inflammatory Mediators

Obesity is a chronic low-grade inflammatory state that is associated with increased risk for the MetS, diabetes and CVD. Indeed the NCEP ATP III identified pro-inflammatory status as being a key MetS characteristic [4]. Previous studies have demonstrated the influence of a variety of pro-inflammatory cytokine polymorphisms, including tumor necrosis factor alpha (*TNFα*) (rs1800629) and interleukin 6 (*IL-6*) (rs1800795 and rs1800797) in the risk of central obesity, diabetes and MetS phenotypes [85,86,87,88,89]. Although results have been inconsistent [90,91], which may be, in part, explained by the fact that as cytokines act in a complex network, single gene activity may not provide full insight into the role of cytokine genes in the MetS. In a recent MetS case control study, Phillips *et al*. examined the relationship between lymphotoxin-α (*LTA*), *IL-6*, and *TNF-α* genetic variants with MetS risk in the LIPGENE-SU.VI.MAX cohort [92]. *TNF-α* rs1800629 major G allele homozygotes and *LTA* rs915654 minor A allele carriers had 20%–40% higher MetS risk [92]. The combined effect of carrying both risk genotypes, which represent half of this population, further increased MetS risk probably attributable to their greater risk of abdominal obesity. Interestingly, this additive effect was further influenced by the presence of *IL-6* rs1800797 G allele. Risk genotype carriers who were also homozygous for the *IL-6* rs1800797 G allele were most at risk of developing the MetS (OR 2.10), fasting hyperglycaemia (OR 2.65) and abdominal obesity (OR 1.52).

Interestingly, IL-6 activates signal transducer and activator of transcription 3 (STAT3), a transcription factor released during the acute-phase response [93]. Common genetic variants at the *STAT3* locus have recently been associated with increased risk of abdominal obesity [94]. As individual genetic variants generally confer only a moderate risk to a trait, analyzing multiple risk alleles simultaneously can be more informative and enhance predictive power, particularly in polygenic conditions. A significant genotype effect between the number of risk alleles and risk of abdominal obesity was identified in the LIPGENE-SU.VI.MAX study, with approximately a 2.5 fold increased risk in individuals carrying two or more risk alleles compared to individuals carrying 1 or no risk alleles [95]. Complement component 3 (C3) is another potential candidate gene with respect to inflammation and diabesity. Elevated concentrations of C3, a protein with a central role in the innate immune system, have been associated with insulin resistance, obesity, the MetS and diabetes [96,97,98,99]. In keeping with previous findings, a dose-dependent relationship between C3 concentrations and the number of MetS components were identified in the LIPGENE-SU.VI.MAX study [100]. Interestingly the increased MetS risk conferred by elevated C3 concentrations (OR 3.11) was abolished in abdominally lean individuals. Furthermore, examination of associations between *C3* polymorphisms and MetS risk demonstrated that common genetic variants at the *C3* locus were associated with risk of the MetS and its phenotypes including dyslipidaemia, abdominal obesity and insulin sensitivity [101]. In recent years adipose tissue has been recognized as an important hormonally active organ. The adipocytokines adiponectin and leptin are thought to play important roles in cardiovascular and metabolic homeostasis. Polymorphisms in the adiponectin (*ADIPOQ*) gene and its receptors (*ADIPOR1*) have been associated with adiponectin levels, insulin resistance, and MetS phenotypes [102,103]. Homozygosity for the leptin receptor (*LEPR*) rs3790433 G allele was also associated with increased MetS risk, which may be accounted for by increased risk of elevated insulin concentrations and insulin resistance [104].

In summary, a major role for genetic susceptibility to obesity, insulin resistance, the MetS and T2DM has been identified. There is no doubt that GWAS in very large populations will rapidly advance our understanding of the genetic basis of these conditions. Indeed the discovery of associated variants in unsuspected genes and outside coding regions illustrates the ability of GWAS to provide potentially important clues into the pathogenesis of these common conditions. Notwithstanding the accumulating evidence regards genetic susceptibility to obesity, T2DM and the MetS, regardless of the approach used, gene-disease-association studies are fraught with difficulties including lack of replication, inadequate statistical power, multiple hypothesis testing, population stratification, publication bias and phenotypic differences. Despite numerous successful discoveries, the effect sizes are small and only explain a fraction of inter-individual variation. Considering the limited success in confirming polygenic variants for diabesity to date it is obvious that more polygenic variants await discovery. Furthermore, in order to further improve our understanding of how these genetic variants interact with environmental factors to modulate disease risk more extensive phenotypic data are required, especially in relation to diet and lifestyle factors.

## 4. Nutrigenetics and Metabolic Disease

According to the “thrifty genotype” hypothesis [105], evolutionary selection of genes (obesity genes) that were originally beneficial for energy storage which conferred a protective effect in times of food deprivation by promoting fat deposition, might, at least in part, explain the current escalating incidence of obesity in a modern Westernized environment of physical inactivity and excessive caloric consumption. This is supported by recent findings that obesity and T2DM reaches epidemic proportions in certain ethnic groups such as Pima Indians, Pacific Islanders, Afro-Americans and Hispanic-Americans [106]. Indeed, the much lower prevalence of metabolic disease observed in Pima Indians in Mexico compared to their counterparts in America illustrates that even in populations who are genetically predisposed to these conditions, their development is largely determined by environmental factors [107,108]. Such data add to the growing body of evidence which suggests that individual’s phenotype represents a complex interaction between genetic and environmental factors over their life course. Nutrition is a key environmental factor in the pathogenesis and progression of common polygenic, diet-related metabolic conditions. The concept of gene-diet interaction describes dietary modulation of the effect of genotype on a particular phenotype (for example obesity, insulin resistance and dyslipidemia) and/or modulation of the effect of a dietary factor on a particular phenotype by a genetic variant. It is generally accepted that the effect of dietary changes on plasma biomarker concentrations differs significantly between individuals. Such inter-individual variability in response to dietary modification is, to a large extent, determined by genetic factors. As discussed already, dietary fat is an important environmental factor and current evidence to support the nutrigenetics concept with respect to obesity, the MetS and T2DM is largely based on data relating to dietary fat [15,32,33]. Other food components such as carbohydrate or fiber can play a role in the development of these conditions. Nevertheless these nutrigenetic investigations provide proof of concept. The *PPARγ* Pro12Ala polymorphism provides an excellent example of the relevance of gene-nutrient interactions in the development of obesity, the MetS and T2DM. In a prospective population-based cohort study, researchers demonstrated an important interaction between habitual dietary fat composition and this SNP [109]. As the ratio of total PUFA to SFA increased a significant inverse relationship was shown for both fasting insulin concentrations and BMI in the Ala carriers, suggesting that the potential protective effect of the Ala allele may be lost in the presence of a high SFA diet. More recently an inverse relationship between Ala frequency and T2DM prevalence has been observed in populations where energy from lipids exceeded 30% of the total energy intake [110]. In recent years a number of well-designed studies (LIPGENE MetS case dietary intervention, LIPGENE-SU.VI.MAX MetS case-control study, Genetics of Lipid Lowering Drugs and Diet Network (GOLDN)) have examined interactions between dietary and/or plasma fatty acid composition and genotype in these diet-related conditions. Some of the key findings from these studies are presented below and summarized in Table 1.

**Table 1:** Gene-nutrient interactions which modulate metabolic syndrome risk.

Gene Locus	Polymorphism	Dietary Factors	Odds Ratio	Conclusions	Reference Number
Acetyl-CoA carboxylase β (*ACC2*)	rs4766587	*n*-6 PUFA	1.82	Risk conferred by the A allele was exacerbated among individuals with a high-fat intake (>35% energy) (OR 1.62), particularly a high intake (>5.5% energy) of *n*-6 PUFA (OR 1.82 for gene-nutrient interaction).	[83]
Apolipoprotein A-I (*APOA1*)	rs670	MUFA	1.57	MetS risk was exacerbated among the habitual high-fat consumers (>35% energy, OR 1.58). In addition a high MUFA fat increased MetS risk (OR 1.57).	[84]
Apolipoprotein B (*APOB*)	rs512535	MUFA	1.89	MetS risk was increased among the habitual high-fat consumers (>35% energy, OR 2.00). Moreover a high MUFA intake increased MetS risk (OR 1.89).	[84]
Complement component 3 ( *C3*)	rs2250656 rs11569562	*n*-6 PUFA	2.2 (rs2250656) 0.32 (rs11569562)	AA genotype for rs2250656 had increased MetS risk relative to minor G subjects. GG genotype for rs11569562 had decreased MetS risk compared with minor A allele carriers.	[101]
Interleukin 1 beta (IL-1β)	6054 G	*n*-3 PUFA	3.29 (GG) 1.95 (GA)	Low *n*-3-PUFA intake (below the median) among the 6054 G allele carriers was associated with increased MetS risk (OR 3.29, for GG and OR 1.95, for GA) compared with the AA genotype.	[88]
Long-chain acyl CoA synthetase 1 (*ACSL1*)	rs9997745	Total PUFA	Risk abolished	GG genotype had increased MetS risk (OR 1.90) compared with the A allele. The risk conferred by GG homozygosity was abolished among those subjects consuming either a low-fat or a high-PUFA diet.	[82]
Leptin receptor (LEPR)	rs3790433	*n*-3 and *n*-6 PUFA	1.65	LEPR rs3790433 GG homozygotes had increased MetS risk (OR 1.65) compared with the minor A allele carriers, which may be accounted for by their increased risk of elevated insulin concentrations (OR 2.40) and insulin resistance (OR 2.15). Low (less than median) plasma (*n*-3) and high (*n*-6) PUFA status exacerbated the genetic risk conferred by GG homozygosity to hyperinsulinemia (OR 2.92–2.94) and insulin resistance (OR 3.40–3.47). These associations were abolished against a high (*n*-3) or low (*n*-6) PUFA background.	[104]
Lymphotoxin-α ( *LTA*) Interleukin 6 (*IL-6*) Tumor necrosis factor-α (*TNF-α*)	*LTA* rs915654 *TNF-α* rs1800629 *IL-6* rs1800797	Total PUFA/SFA	4.4	*LTA* rs915654 minor A allele carriers and *TNF-α* rs1800629 major G allele homozygotes had increased MetS risk (OR 1.37 and OR 1.35). Possession of the *IL-6* rs1800797 GG genotype by the *LTA* and *TNF-α* “risk genotype” carriers further increased MetS risk (OR 2.10). Low total PUFA/SFA exacerbated MetS risk (OR 4.4).	[92]
Peroxisome proliferator-activated receptor-delta (PPAR-δ)	-87T>C	Total fat	0.42	Low dietary fat consumers (<34.4% of energy from fat (median of fat consumption)) carrying the -87C allele had reduced MetS risk (OR 0.42).	[111]
Transcription factor 7-like 2 (TCF7L2)	rs7903146	Total SFA	2.35	High SFA intake (≥15.5% energy) exacerbated MetS risk (OR 2.35) and was associated with further impaired insulin sensitivity in the T allele carriers of rs7903146 compared to the CC homozygotes and particularly to the T allele carriers with the lowest SFA intake.	[60]

Metabolic Syndrome (MetS); monounsaturated fatty acids (MUFA); polyunsaturated fatty acids (PUFA); saturated fatty acids (SFA) Adapted from Perez-Martinez *et al*. [31].

### 4.1. TCF7L2

Data from the Diabetes Prevention Program and the Diabetes Prevention Study indicate that lifestyle or environmental factors can modulate the genetic effects of TCF7L2 polymorphisms [47,54]. In the Diabetes Prevention Study overweight individuals with impaired glucose tolerance were allocated to an intensive diet and lifestyle intervention group or a control group. After a mean 4-year follow-up period they found that TCF7L2 polymorphisms were associated with the incidence of diabetes in the control group, but not the intervention group, suggesting that environmental factors can reduce genetic susceptibility even when risk genotypes are related to impaired insulin secretion. In the GOLDN study total dietary PUFA modulated the genetic effects of the TCF7L2 rs7903146 polymorphism on postprandial lipemia [56]. In the LIPGENE dietary intervention study, TCF7L2SNPs were associated with plasma lipid concentrations, carbohydrate metabolism, blood pressure and inflammatory markers. Interactions with total SFA were noted. For example, among the rs11196224 major homozygotes elevated plasma SFA was associated with increased insulin resistance [112]. Similarly in the LIPGENE-SU.VI.MAX study rs7903146 was associated with increased MetS risk, arising from their impaired insulin sensitivity, greater insulin resistance, increased abdominal obesity and hypertension [60]. Interestingly dietary fat intake, recorded 7.5 years prior to MetS case/control selection, modulated the genetic influence on MetS risk. In particular high dietary SFA intake (≥15.5% of energy) accentuated the deleterious effects of rs7903146 on MetS risk, suggesting that the long-term effect of dietary fatty acid composition and consumption may have the potential to modify the genetic susceptibility of developing the MetS. 

### 4.2. FTO

Limited cross-sectional analysis of the influence of dietary factors on BMI according to *FTO* rs9939609 genotype indicates that high-fat diets increase obesity risk [113,114]. However, these studies did not investigate specific effects of dietary fat type or fatty acid composition. Recent data from a study of 354 children identified an interaction between dietary SFA and the ratio of total PUFA to SFA and obesity associated with *FTO* rs9939609 [115]. In the LIPGENE-SU.VI.MAX study *FTO* rs9939609 was associated with increased risk of having a BMI in the overweight or obese category and of being abdominally obese [116]. Increased obesity risk was maintained over the 7.5 year follow-up period and while rs9939609 was not associated with MetS risk, risk of these obesity related measures was higher in the risk allele carrying MetS cases relative to their non-risk allele carrying counterparts. A novel finding in that study was that high habitual dietary SFA consumption (≥15.5% of energy) and low PUFA:SFA accentuates obesity risk in the *A* allele carriers but not in the *TT* homozygotes in this adult European population, suggesting that genetic predisposition to obesity may be modulated by dietary SFA intake. This may be particularly relevant to individuals with diet-related metabolic disease who are at increased cardiometabolic risk. Recent data from the GOLDN study also identified an interaction between rs9939609 and SFA intake, whereby homozygous participants in this American population had a higher BMI only when they had a high SFA intake (>mean) [117]. Interestingly, hypothalamic *FTO* over-expression has been shown to result in a 4-fold increase in *STAT3* expression [118]. Given the importance of STAT3 in the leptin signaling pathway these data suggest a potential mechanism for mediating *FTO*’s actions and potential modulation by SFA.

### 4.3. Fatty Acid and Lipid Metabolism Genes

Acetyl-CoA carboxylase β (ACC2) plays a key role in fatty acid synthesis and oxidation pathways, disturbance of which is associated with impaired insulin sensitivity and MetS. The LIPGENE-SU.VI.MAX study examined whether several *ACC2* polymorphisms (rs2075263, rs2268387, rs2284685, rs2284689, rs2300453, rs3742023, rs3742026, rs4766587, and rs6606697) influence MetS risk, and whether dietary fatty acids modulate this interaction [83]. Minor A allele carriers of rs4766587 had increased MetS risk (OR 1.29) compared with the GG homozygotes, which may in part be explained by their increased BMI, abdominal obesity, and impaired insulin sensitivity. Dietary fat intake modulated MetS risk such that risk conferred by the A allele was exacerbated among individuals with a high-fat intake (>35% energy) (OR 1.62). Conversely MetS risk was abolished among individuals with a low-fat intake. Examination of individual fatty acid classes identified a gene-nutrient interaction with PUFA intake, whereby A allele carriers with high PUFA intake (>5.5% energy) had increased MetS risk (OR 1.53). This gene-nutrient interaction was reflected by both *n*-6 (OR 1.80) and *n*-3 PUFA (OR 1.75). Importantly, some of these findings were replicated in an independent cohort (LIPGENE MetS only dietary intervention cohort). Thus, the genetic associations with increased BMI, body-weight, waist circumference, and insulin resistance were confirmed. Consistent with the original findings, these genetic differences persisted in the high-fat but not among the low-fat consumers. In summary, genetic variation at the *ACC2* gene locus influences MetS risk, which was modulated by dietary fat.

Long-chain acyl CoA synthetase 1 (ACSL1) is important for mitochondrial beta-oxidation of long chain fatty acids and plays an important role in fatty acid metabolism. Disturbance of these pathways may result in insulin resistance and dyslipidemia, key MetS features [119,120,121]. Examination of the relationship between *ACSL1* polymorphisms (rs4862417, rs6552828, rs13120078, rs9997745, and rs12503643) and MetS risk and potential interactions with dietary fat conducted in the LIPGENE-SU.VI.MAX study [82]. Subjects with the GG genotype for rs9997745 SNP had increased MetS risk (OR 1.90), displayed elevated fasting glucose and insulin concentrations and increased insulin resistance compared with subjects carrying the A allele. Moreover MetS risk was modulated by dietary fat, whereby the risk conferred by GG homozygosity was effectively abolished among those subjects consuming either a low-fat or when total dietary PUFA intake was in the top 50th percentile. Examination of the HAPMAP data for this SNP indicates allele frequency differences between ethnic groups. Whereas the allele frequency in the LIPGENE-SU.VI.MAX study is not far from that in the European HAPMAP population, the opposite is true in Africans where the G allele is the minor allele. It is quite interesting to note that this SNP is not polymorphic in Asians. This implies that in an Asian population everybody will carry the “risk” allele in the presence of a total PUFA-poor diet (which has been the traditional diet in Asian countries) and may be particularly at risk of MetS. In conclusion, *ACSL1* rs9997745 influences MetS risk, most likely via disturbances in fatty acid metabolism, which was modulated by dietary fat consumption.

APOA1 is the major protein component of HDL and also is an activator of the enzyme lecithin-cholesterol acyltransferase (LCAT), a key component of reverse cholesterol transport. In contrast, plasma APOB, the main component of LDL, is essential for the assembly and secretion of the triglyceride-rich lipoproteins. Several SNPs at these loci have been proposed to influence MetS risk. In the LIPGENE-SU.VI.MAX study, the *ApoB* rs512535 and *ApoA1* rs670 major G allele homozygotes had increased MetS risk (OR 1.65 and OR 1.42, respectively), which may be explained by their increased abdominal obesity and impaired insulin sensitivity but not dyslipidemia [84]. These associations derived primarily from the male GG homozygotes (*ApoB* rs512535 OR 1.92 and *ApoA1* rs670 OR 1.50). On the other hand, MetS risk was exacerbated among the habitual high-fat consumers (>35% energy) (*ApoB* rs512535 OR 2.00 and OR 1.58 for *ApoA1* rs670). In addition a high dietary MUFA intake increased MetS risk (OR 1.89 and OR 1.57 for *ApoB* rs512535 and *ApoA1* rs670, respectively). MetS risk was diminished among the habitual low-fat consumers (<35% energy). In summary *ApoB* and *ApoA1* polymorphisms may influence MetS risk. Modulation of these associations by gender and dietary fat suggests novel gene-gender-diet interactions. As already alluded to in section 3.4, the postprandial period, which is the most metabolically abnormal, is of particular importance as humans spend most of their lives in this phase. During which circulating lipoproteins are involved in a cascade of changes to their composition and concentration. It is generally accepted that the impact of dietary fat on postprandial lipoprotein response differs between individuals, and genetic factors are thought to be one of the key determinants of such inter-individual variability. Nutrigenetics of postprandial lipid metabolism including evidence from human dietary interventions has recently been reviewed [122,123]. 

### 4.4. Pro-Inflammatory Cytokines, Adipocytokines and Inflammatory Mediators

As already eluded to, inflammation plays a key role in insulin resistance. We have shown that total plasma PUFA/SFA levels modified the observed additive genetic effects of *IL-6*, *TNFα* and *LTA* [92]. When stratified according to median plasma PUFA/SFA levels, MetS risk was four-fold higher in the 3 SNP risk genotype carriers with the lowest PUFA/SFA levels compared to non-carriers and was thought to be driven by the SFA content, with high SFA levels alone accounting for 5-fold increased MetS risk. A low PUFA/SFA ratio also exacerbated their increased risk of several phenotypes (abdominal obesity, fasting hyperglycaemia, hypertension and pro-inflammatory profile). Interestingly, when risk genotype carriers with the lowest PUFA/SFA levels were compared with their risk genotype carriers with the highest PUFA/SFA levels significant improvements to their metabolic profile were noted. Most importantly a high PUFA/SFA ratio attenuated genetic predisposition to MetS risk. Moreover, risk genotype carriers with the highest PUFA/SFA levels had reduced pro-inflammatory status, lower TAG levels and HOMA-IR values than risk genotype carriers with the lowest PUFA/SFA levels. These findings support current guidelines to reduce dietary SFA intake and increase PUFA consumption.

Investigation of the effect of *ADIPOQ* SNPs, rs266729 and rs17300539, on metabolic-related traits, and their modulation by dietary fat in white Americans in the GOLDN study revealed significant interaction with MUFA intake [124]. In subjects with high MUFA intake (>median) rs17300539 A allele carriers had lower BMI and decreased obesity risk. Dietary intervention analysis has demonstrated that CC homozygotes for rs266729 were less insulin resistant after consumption of MUFA and carbohydrate rich diets compared to the SFA rich diet [125]. Furthermore in the LIPGENE dietary intervention study two SNPs (rs266729 in *ADIPOQ*, and rs10920533 in *ADIPOR1*) interacted with plasma SFAs to alter insulin and HOMA-IR [126]. This study demonstrated that a reduction in plasma SFA decreased insulin resistance in carriers of the minor allele of rs266729 *ADIPOQ* and rs10920533 *ADIPOR1*. Personalised nutrition advice based on this data would recommend a decrease in SFA consumption in the diet of MetS subjects carrying the minor allele of rs266729 *ADIPOQ* and/or rs10920533 *ADIPOR1*. 

The LEPR is an adipocytokine receptor that is involved in the regulation of fat metabolism and has been associated with insulin resistance, a key feature of MetS. In the LIPGENE-SU.VI.MAX study GG homozygotes of rs3790433 SNP at the *LEPR* gene had increased MetS risk compared with the minor A allele carriers (OR 1.65), which may be accounted for by their increased risk of elevated insulin concentrations and insulin resistance [104]. Low (less than median) plasma *n*-3 and high *n*-6 PUFA status exacerbated the genetic risk conferred by GG homozygosity to hyperinsulinemia (OR 2.92–2.94) and insulin resistance (OR 3.40–3.47). Interestingly, these associations were abolished against a high *n*-3 or low *n*-6 PUFA background. Importantly, these findings were replicated in an independent MetS cohort. Homozygosity for the *LEPR* rs3790433 G allele was associated with insulin resistance, which may predispose to increased MetS risk. Of note, significant improvements to indices of insulin sensitivity and insulin resistance by the GG homozygotes were identified following a 12 weeks low-fat dietary intervention supplemented with *n*-3 long chain (LC) PUFA. These data suggest that genetic influences associated with this *LEPR* polymorphism may be selectively modulated by *n*-3 LC-PUFA. In conclusion these data from the LIPGENE project suggest novel gene-nutrient interactions whereby the deleterious effects associated with LEPR rs3790433 GG homozygosity were more evident against a background of low *n*-3 or high *n*-6 PUFA, and to a lesser extent with high SFA status. As *LEPR* rs3790433 GG homozygotes appear to be sensitive to plasma and dietary fatty acid composition, these individuals may derive the most benefit from dietary manipulation and current guidelines to reduce dietary SFA and increase *n*-3 PUFA intake.

The LIPGENE-SU.VI.MAX study also identified a gene-nutrient interaction between *STAT3* polymorphisms with SFA. High dietary SFA intake (≥15.5% of energy) modulated the genetic association between *STAT3* polymorphisms with obesity [94]; carriers of more than 2 risk alleles with the highest SFA consumption further increased their risk of abdominal obesity by 32% compared to those carrying one or fewer risk alleles. This data suggests that individuals with certain STAT3 genotypes are more sensitive to SFA and that these individuals may derive the most benefit from dietary manipulation and current guidelines to reduce dietary SFA intake. While the mechanisms underlying these findings are unknown, it is possible that toll-like receptor-4, the molecular link between fatty acids, obesity, inflammation and insulin resistance [127] may play a role. Interestingly, hypothalamic FTO over-expression has been shown to result in a 4-fold increase in *STAT3* expression [118]. Given the importance of STAT3 in the leptin signaling pathway these data suggest a potential mechanism for mediating FTO’s actions and potential modulation by SFA. 

The increased MetS risk associated with the *C3* rs2250656 A allele in the LIPGENE-SU.VI.MAX study may be explained by their classic MetS profile and raised inflammatory status [101]. Interestingly plasma PUFA modified MetS risk whereby the combination of carrying two “risk” A alleles and having low *n*-6 or total PUFA (below the median) exacerbated MetS risk, suggesting that these individuals, who represent approximately half of the population and who are genetically predisposed to the MetS, are also more sensitive to PUFA. Similarly reduced MetS risk associated with the rs11569562 polymorphism was subject to a significant effect modification by PUFA, with the greatest protection from the MetS being achieved by GG homozygotes with the highest total PUFA status [101]. Likewise GG homozygotes with the highest LC *n*-3 PUFAs had the lowest risk of hypertriglyceridaemia. In keeping with these findings, in the LIPGENE dietary intervention study the “protective” rs11569562 GG genotype was associated with enhanced insulin sensitivity and these individuals were more responsive to LC *n*-3 PUFA, compared to the A allele carriers. Following a 12 weeks low-fat (28% energy), high-complex carbohydrate diet intervention supplemented with 1.24 g/day LC *n*-3 PUFA, GG homozygotes displayed beneficial changes to their lipid profile (10% reduction in NEFA, 8% non-significant reduction in TAG, 5% reduction in total cholesterol and 17% reduction in LDL concentrations), compared to the A allele carriers. No changes were observed between genotypes when subjects on the same diet received a 1 g/day high oleic acid control supplement. In addition, the “at risk” rs2250656 A allele carriers had reduced insulin sensitivity and increased BMI, relative to the GG homozygotes. Again, genetic influences were modified by LC *n*-3 PUFA supplementation, whereby the A allele carriers achieved a 35% improvement in insulin sensitivity following intervention whereas no changes were noted between genotypes following oleic acid supplementation. Interestingly PUFAs are ligands of FXR [128], a nuclear receptor which regulates *C3* expression [129]. Thus it is possible that alteration of *C3* expression via modulation of FXR is a potential mechanism by which gene-nutrient interaction of *C3* genotype and dietary PUFAs could influence C3 levels and thus MetS risk. Although speculative, it may be worthy of further investigation to help elucidate the molecular basis of such gene-nutrient interactions and their impact on markers of inflammation and insulin resistance.

## 5. Nutrigenetics and Personalised Nutrition

Phenylketonuria (PKU) was the first genetic disease in which a gene-diet interaction was described. This condition is a good example of how a single nutrient can be used to manage genetic predisposition to a monogenic disease. People with PKU lack the enzyme required to metabolize phenylalanine, an essential amino acid found in dairy, meat, fish, nuts and pulses, with the result that dangerous levels of phenylpyruvic acid may build up which are toxic to the brain Thus, individuals with PKU need to stick to a low phenylalanine diet for life to avoid PKU symptoms. Coeliac disease, an inflammatory condition which results from intolerance to dietary gluten, is an example of how personalised nutrition can potentially work. High concordance rates from twin studies indicate a strong genetic influence, but it seems that carrying certain genes reveals a genetic predisposition to dietary factors rather than disease development [130]. Obesity is another example of how nutrigenetics can be used to personalise an individual’s diet with a view to improving long term weight management. An interesting study by Arkadianos *et al*. [131] examined weight loss and weight loss maintenance following a personalised calorie-controlled diet and exercise program, based on 24 SNPs in 19 genes involved in metabolism, in subjects with a history of weight loss failure compared to control subjects who just received generic dietary and exercise advice. This study showed that the nutrigenetically tailored diet achieved better compliance, improvements in glucose levels and BMI reduction not only during the weight loss period but importantly also over the following year. Another personalised dietary intervention, based on 4 SNPs in four genes, with stratification of overweight/obese and control subjects into diet or diet and exercise groups, demonstrated that individuals were slow to take optimal health advice, particularly in the combined diet and exercise group [132]. While this was a small study based on a limited number of genetic variants it raises the issue of negative consumer opinion, which poses a potential barrier to the application of nutrigenetic based intervention. A recent pan-European study investigated the attitudes of consumers towards genetic testing and personalised nutrition [133]. The results of this study were encouraging, with 66% of respondents willing to undergo genetic testing and 27% willing to adhere to a personalised diet [133]. Interestingly individuals with MetS and T2DM related health conditions were particularly positive toward nutrigenetic intervention. These findings are encouraging for the future application of genome-customized diets for obesity, MetS and T2DM prevention and therapy following personalised approaches. However, as success or failure of any new technology is consumer driven, consumer research in the application of personalised nutrition is essential.

## 6. Conclusions

In this review, some recent novel nutrigenetic data in the context of metabolic disease have been presented, which suggest that certain nutrients, in particular dietary fatty acids, may have the potential to modify the genetic predisposition to these diet-related conditions. While this review has focused on dietary fat, more holistic methods which incorporate an individuals’ diet or dietary patterns, rather than selecting individual dietary components, need to be developed to advance the state of the art. Moreover, other modifiable environmental factors which interact with the diet should be considered in gene-environment studies (*i.e.*, physical activity, alcohol intake, smoking status) across a range of metabolic conditions. Nevertheless current data provides proof of concept. The shift towards “personalised” nutritional advice is an attractive proposition. Nutrigenetics has the potential to change diet-related disease prevention and therapy. While recent advances in high-throughput genetic analysis have improved our understanding of the contribution of genetics to metabolic health and disease, the molecular mechanisms underlying many of these gene-nutrient interactions remain unclear. Functional studies are needed to ascertain their biological significance and potential clinical utility. Nutrigenetics is just one piece in a very complex jigsaw, which needs to move forward with nutritional science in order to translate observational findings into molecular mechanisms. The combined application of nutritional and genetic epidemiology with metabolite and molecular profiling at the gene, transcriptome, proteome and metabolome level to define an individuals’ metabotype will be crucial in this regard. Such concerted actions, using larger study cohorts and collaborative research efforts across different disciplines may lead to the identification of sensitive/responsive metabotypes (*i.e.*, modifiable by dietary fatty acids or other nutrients). The challenge for current and future research is validation and translation of nutrigenetic findings, which may provide the basis for successful personalised and public health approaches for metabolic disease prevention.
